# Supplementary material for: Characterization of a Novel Splicing Variant in Acylglycerol Kinase (AGK) Associated with Fatal Sengers Syndrome
Source: Int J Mol Sci. 2021 Dec 15;22(24):13484. doi: 10.3390/ijms222413484 (PMC8708263; doi:10.3390/ijms222413484)
Supplement: Supplementary file 1 [file ijms-22-13484-s001.zip › ijms-1495764-supplementary.pdf]

Table S1. Design of mitochondrial multi-gene panel (MITO panel), consisting of a group of genes previously associated with mitochondrial diseases.

MITO panel
AARS2, ABCB11, ACAD8, ACAD9, ACO2, ADCK1, ADCK2, ADCK3, ADCK4, ADCK5, ADSL, AFG3L2, AGK, AIFM1, AKR1B15, APOPT1, APTX, ATAD1, ATAD3A, ATP5A1, ATP5B, ATP5C1, ATP5D, ATP5E, ATP5F1, ATP5F1D, ATP5G1, ATP5G2, ATP5G3, ATP5O, ATPAF1, ATPAF2, BCS1L, BOLA3, C10ORF2, C12ORF65, C19ORF12, C1QBP, CA5A, CARS2, CCDC88A, CEP89, CHCHD10, CLPB, CLPP, COA5, COA6, COA7, COQ10A, COQ10B, COQ2, COQ3, COQ4, COQ5, COQ6, COQ7, COQ9, COX10, COX11, COX14, COX15, COX16, COX17, COX18, COX19, COX4I1, COX4I2, COX6A1, COX6A2, COX6B1, COX6B2, COX7B, COX8A, CPS1, CYC1, CYCS, DARS2, DEAF1, DGUOK, DLAT, DLD, DNA2, DNAJC19DNM1L, E4F1, EARS2, ECHS1, ELAC2, ETHE1, FAM36A, FARS2, FARSB, FASTKD2, FBXL4, FDX1L, FH, FLAD1, FOXRED1, GARS, GFER, GFM1, GFM2, GLRX5, GOT2, GPT2, GTPBP3, GYG2, HARS2, HCCS, HIBCH, HSD17B10, HSD17B4, HSPD1, HSPE1, HTRA2, IARS, IARS2, IBA57, ISCA1, ISCA2, ISCU, ITPA, KARS, LARS2, LETM1, LGI1, LIAS, LIPT1, LIPT2, LONP1, LRP4, LRPPRC, LYRM4, LYRM7, MARS2, MDH2, MECP2, MFF, MFN1, MFN2, MGME1, MICU1, MIPEP, MNF1, MPV17, MRPL12, MRPL3, MRPL44, MRPS16, MRPS22, MRPS23, MRPS34, MRPS7, MTCH1, MTERF1, MTFMT, MTHFD1L, MTO1, MTPAP, NADK2, NARS2, NAT8L, NAXE, NDUFA1, NDUFA10, NDUFA11, NDUFA12, NDUFA13, NDUFA2NDUFA3, NDUFA4, NDUFA4L2, NDUFA5, NDUFA9, NDUFAF1, NDUFAF2, NDUFAF3, NDUFAF4, NDUFAF5, NDUFAF6, NDUFB1, NDUFB10, NDUFB11, NDUFB2, NDUFB3, NDUFB4, NDUFB5, NDUFB6, NDUFB7, NDUFB8, NDUFB9, NDUFS1, NDUFS2, NDUFS3, NDUFS4, NDUFS5, NDUFS6, NDUFS7, NDUFS8, NDUFV1, NDUFV2, NDUFV3, NFS1, NFU1, NUBPL, OPA1, OPA3, PARS2, PC, PCK2, PDHA1, PDHA2, PDHB, PDHX, PDK3, PDP1, PDP2, PDSS1, PDSS2, PET100, PET117, PGAP2, PIGN, PMPCB, PNPLA4, PNPLA8, PNPT1, POLG, POLG2, POLRMT, PTCD1, PUS1, QRSL1, RANBP2, RARS2, RERE, RMND1, RNASEH1, RPIA, RRM2B, RTN4IP1, SARS, SARS2, SCO1, SCO2, SDHA, SDHAF1, SDHAF2, SDHB, SDHC, SDHD, SERAC1, SFXN4, SLC19A3, SLC25A12, SLC25A19, SLC25A26, SLC25A3, SLC25A4, SLC25A42, SLC25A46, SLC52A2, SLC6A8, SLC6A9, SPG20, SPG7, SUCLA2, SUCLG1, SURF1, TACO1, TANGO2, TARS2, TAZ, TAZ, TBCD, TBCE, TFAM, TIMM8A, TK2, TKT, TMEM126A, TMEM126B, TMEM70, TOP3A, TPK1, TRAK1, TRIT1, TRMT10C, TRMT5, TRMU, TRNT1, TSFM, TTC19, TUBB, TUBB6, TUFM, TXN2, TYMP, UPB1, UQCC3, UQCR10, UQCR11, UQCRB, UQCRC1, UQCRC2, UQCRFS1, UQCRH, UQCRHL, UQCRQ, VAC14, VARS2, VDAC1, VDAC2, WARS2, YARS2, YME1L1

Table S2. Parameters calculated to assess mitochondrial function, based on oxygen consumption rate (OCR), and glycolytic function, based on extracellular acidification rate (ECAR).

a)

PARAMETER	DESCRIPTION
**BASAL RESPIRATION**	Baseline mitochondrial respiration before addition of compounds
**ATP SYNTHESIS**	Oligomycin-sensitive respiration: basal respiration fraction used for ATP production
**PROTON LEAK**	Oligomycin-insensitive respiration: refers to the remaining basal respiration not coupled to ATP production
**COUPLING EFFICIENCY**	Ratio of ATP turnover-linked respiration and basal respiration: an indicator of OXPHOS and electron chain coupling quality
**MAXIMAL RESPIRATION**	FCCP: an uncoupler that disrupts ATP synthesis by transporting protons across mitochondrial inner membranes and depolarizing mitochondrial membrane potential. This will reflect the maximal capacity of the ETC
**SPARE RESPIRATORY CAPACITY**	Difference between the maximal respiration after FCCP and basal respiration: this indicates the ability of the cell to respond to an energy demand
**NON-MITOCHONDRIAL RESPIRATION**	Final OCR measurement after the addition of Rot/Ant

b)

PARAMETER	DESCRIPTION
**GLYCOLYSIS RATE**	Process which converts glucose into pyruvate. It is measured as the rate of ECAR reached by the cells after glucose addition
**GLYCOLYTIC CAPACITY**	Maximum rate of ECAR reached after the addition of olygomycin, which inhibits OXPHOS, forcing the cells to use glycolysis at maximum capacity
**Glycolytic Reserve**	Difference between the maximum glycolytic capacity and glycolysis, reflecting the cells' ability to respond to an energy demand

Table S3. In silico analysis to predict *AGK* variant pathogenicity according to AMCG guidelines.

Variants AGK (NM_018238.4)	Inheritance	Population frequencies			Conservation Scores			Prediction Scores			Splicing	
		gnomAD	ExAC	1000 Genomes	GERP	PhyloP100way	PhastCons100way	DANN	Mutation Taster	FATHMM	dbscSNV	HSF
c.518+1G>A	Mother Father	-	-	-	5.20	7.392	1.000	0.9955	DC	D	0.9999	Broken WT Donor site

DC: Disease causing; D: Damaging; WT: Wild type

Table S4. Genetic and clinical findings in patients with Sengers syndrome.

Patient	Genetic Analysis			Clinical Course						References
	Variants AGK (NM_018238.3)	Variants AGK (NP_060708)	Variant	Age at Onset	Course	OXPHOS Defect	Cardiomyopath y	Plasma Lactic Acidosis	Cataracts	
P1	c.3G>C c.517C>T	p.Met1Ile p.Gln173*	Missense Nonsense	3 m	Alive at 36 y	ND	YES	YES	YES	Lalive d'Epinay et al. 1986
P2	c.3G>C c.672C>A	p.Met1Ile p.Tyr224*	Missense Nonsense	3 m	Alive at 35 y	-	YES	Exercise	YES	Lalive d'Epinay et al. 1986
P3	c.1131+5G>A c.1131+5G>A	-	Splicing defect	3.5 y	Alive at 41 y	-	YES	YES	YES	van Ekeren,, et al. 1993

P4	c.1131+5G>A<br>c.1131+5G>A	-	Splicing defect	1 y	Death at 12 y	I, II+III, IV, V	YES	NO	YES	Morava et al. 2004
P5	c.1131+5G>A<br>c.1131+5G>A	-	Splicing defect	Birth	Alive at 10 y	I, II+III, IV, V	YES	YES	YES	Morava et al. 2004
P6	c.221+1G>A<br>c.1213C>T	-<br>p.Gln405*	Splicing defect Nonsense	10 m	Alive at 12 y	I, II, III, IV	YES	YES	YES	Di Rosa et al. 2006
P7	c.306C>T<br>c.841C>T	p.Tyr102*<br>p.Arg281*	Nonsense	1 w	Death at 18 d	I, II+III, IV, V	YES	YES	YES	Mayr et al. 2012
P8	c.412C>T<br>c.1137_1143del	p.Arg138*<br>p.Gly380Leufs*16	Nonsense Frameshift	1 w	Death at 11 m	-	YES	Exercise	YES	Mayr et al. 2012
P9	c.672C>A<br>c.870del	p.Tyr224*<br>p.Gln291Argfs*8	Nonsense Frameshift	Birth	Death at 10 m	I, II, III, IV, V	YES	YES	YES	Mayr et al. 2012
P10	c.101+?_222-?del<br>c.101+?_222-?del		Deletion	4 m	Death at 8 m	I, II, III, IV, V	YES	YES	YES	Mayr et al. 2012
P11	c.297+2T>C<br>c.1170T>A	p.Lys75Glnfs*12<br>p.Tyr390*)	Frameshift	<1 y	Death at 18 y	I, III, IV	YES	YES	YES	Calvo et al. 2012
P12	c.1131+1G>T<br>c.1131+1G>T	p.Ser350Glufs*19	Frameshift	Birth	Death at 4 d	I, III, IV	ND	YES	YES	Calvo et al. 2012
P13	c.424-3C>G<br>c.424-3C>G	p.Ala142Thrfs*4	Frameshift	Birth	Alive at 17 y	-	-	-	YES	Aldahmesh et al. 2012
P14	c.424-3C>G<br>c.424-3C>G	p.Ala142Thrfs*4	Frameshift	Birth	Alive at 11 y	-	-	-	YES	Aldahmesh et al. 2012
P15	c.424-3C>G<br>c.424-3C>G	p.Ala142Thrfs*4	Frameshift	Birth	Alive at 7 y	-	-	-	YES	Aldahmesh et al. 2012
P16	c.979A>T<br>c.979A>T	p.Lys327*	Nonsense	Birth	Dead 5 m		YES	YES	YES	Siriwardena et al. 2014

P17	c.979A>T<br>c.979A>T	p.Lys327*	Nonsense	Birth	Dead<br>12 d	I, I+III, II+III, III, IV	YES	YES	YES	Siriwardena et al. 2014
P18	c.979A>T<br>c.979A>T	p.Lys327*	Nonsense	Birth	Dead<br>2 d	ND	YES	ND	YES	Siriwardena et al. 2014
P19	c.979A>T<br>c.979A>T	p.Lys327*	Nonsense	Birth	Dead<br>18 d	ND	YES	ND	YES	Siriwardena et al. 2014
P20	c.3G>A<br>c.3G>A	p.Met1	Missense	Birth	Dead<br>6 m	ND	YES	YES	YES	Siriwardena et al. 2014
P21	c.3G>A<br>c.3G>A	p.Met1	Missense	2 m	Alive at<br>2 y	ND	YES	-	YES	Siriwardena et al. 2014
P22	c.523_524delAT<br>c.523_524delAT	p.Ile175Tyrfs*2	Nonsense<br>Frameshift	5 m	Death at<br>7 m	ND	YES	YES	YES	Haghighi et al. 2014
P23	c.424-1G>A<br>c.424-1G>A	-	Splicing defect	Birth	Death at<br>10 d	ND	YES	YES	YES	Haghighi et al. 2014
P24	c.424-1G>A<br>c.424-1G>A	-	Splicing defect	Birth	Death at<br>4 m	ND	YES	YES	YES	Haghighi et al. 2014
P25	c.409C>T<br>c.409C>T	p.Arg137*	Nonsense	NA	Death at<br>3 m	I	YES	YES	NA	Haghighi et al. 2014
P26	c.409C>T<br>c.409C>T	p.Arg137*	Nonsense	Birth	Death at<br>6 m	I	YES	YES	YES	Haghighi et al. 2014
P27	c.871C>T<br>c.1035dup	p.Gln291*<br>p.Ile346Tyrfs*39	Nonsense<br>Frameshift	Birth	Alive at<br>3 m	ND	YES	YES	YES	Haghighi et al. 2014
P28	c.297+2T>C<br>c.841C>T	p.Lys75Glnfs*12<br>p.Arg281*)	Frameshift<br>Nonsense	Birth	Alive at<br>10 y	I	YES	-	YES	Haghighi et al. 2014
P29	c.877+3G>T<br>c.877+3G>T	-	Splicing defect	Birth	Alive at<br>15 y	ND	YES	-	YES	Haghighi et al. 2014
P30	c.297G>T<br>c.297G>T	p.Lys99Asg	Missense	5 d	Death at<br>22 m	ND	ND	ND	ND	Deniz Kor et al. 2016
P31	c.412C>T<br>c.412C>T	p.Arg138*	Nonsense	23 d	Death at<br>3 m	ND	ND	ND	ND	Deniz Kor et al. 2016
P32	c.979A>T<br>c.979A>T	p.Lys327*	Splicing defect	1 d	Death at<br>1 d	ND	YES	YES	ND	Beck et al. 2018
P33	c.1047-2A>T	-	Splicing	1 y	Death at	ND	YES	YES	YES	Das B et al. 2019

	c.1047-2A>T		defect		13 y					
P34	c.1215dupG<br>c.1215dupG	p.Phe406Valfs4	Frameshift	3 m	Death at 9 m	ND	ND	ND	ND	Guleray Naz et al. 2019
P35	c.518+1G>A<br>c.518+1G>A	-	Splicing defect	Birth	Death at 24 h	I, V	YES	YES	YES	Present study

h: hour; d: days; m: month; y: year; ND: Not described.
